# Urinary Incontinence Among Elite Track and Field Athletes According to Their Event Specialization: A Cross-Sectional Study

**DOI:** 10.1186/s40798-022-00468-1

**Published:** 2022-06-15

**Authors:** Elena Sonsoles Rodríguez-López, María Barbaño Acevedo-Gómez, Natalia Romero-Franco, Ángel Basas-García, Christophe Ramírez-Parenteau, Sofía Olivia Calvo-Moreno, Juan Carlos Fernández-Domínguez

**Affiliations:** 1 Physiotherapy Department, Universidad Camilo José Cela, 28692 Madrid, Spain; 2 Physiotherapy Department, Spanish Triathlon Federation, Madrid, Spain; 3 Nursing and Physiotherapy Department, University of the Balearic Islands, Road to Valldemossa, km 7.5, 07122 Palma, Spain; 4 Health Research Institute of the Balearic Islands (IdISBa), Palma, Spain; 5 Physiotherapy Department, Royal Spanish Athletics Federation, Madrid, Spain; 6 Medical Department, Royal Spanish Athletics Federation, Madrid, Spain

**Keywords:** Sports, Pelvic floor disorders, Athletic performance

## Abstract

**Background:**

Physical effort in sports practice is an important trigger for urinary incontinence (UI). Among high-impact sports, all track and field events require continuous ground impacts and/or abdominal contractions that increase intra-abdominal pressure and impact on the pelvic floor musculature. However, studies to date have not taken into account the specific sports tasks that elite track and field athletes perform according to the competitive events for which they are training.

**Methods:**

This cross-sectional study describes the prevalence, type, and severity of UI among elite track and field athletes considering their event specialization and training characteristics. A total of 211 female and 128 male elite track and field athletes answered an online questionnaire including anthropometric measures, medical history, training characteristics, and UI symptoms. To determine self-reported UI, the International Consultation on Incontinence Questionnaire-UI Short-Form (ICIQ-UI-SF) was used. To determine UI type and severity, the incontinence questionnaire and incontinence severity index were used, respectively.

**Results:**

The ICIQ-UI-SF showed that 51.7% of female and 18.8% of male athletes had UI, with stress UI (SUI) being the most frequent type (64.4%) for female and urge UI for male athletes (52.9%). Of athletes who were not identified as having UI according to the questionnaires, 24.6% of female and 13.6% of male athletes experienced urine leakage during training, mainly during jumping. Although training characteristics (experience, volume, and resting) were not related to UI, female athletes specializing in vertical jumps showed significantly lower UI prevalence compared to those specializing in horizontal jumps (*χ*^2^ [1] = 4.409, *p* = 0.040), middle-distance running (*χ*^2^ [1] = 4.523, *p* = 0.033), and sprint/hurdles events (*χ*^2^ [1] = 4.113, *p* = 0.043). These female athletes also displayed the lowest training volume. No differences were shown for males (*p* > 0.05).

**Conclusions:**

Over half of the elite track and field female athletes have self-reported UI, especially SUI, and prevalence is higher when considering urine leakage events during training. Training characteristics and specialization were not related to UI identified by questionnaires, but female athletes specializing in vertical jump events showed the lowest prevalence and training volume. Males showed significantly lower prevalence, without correlation with their specialization. Sport professionals should increase UI detection among elite athletes and design-specific approaches that consider their physical demands to make visible, prevent, or improve pelvic floor dysfunction in this population.

**Supplementary Information:**

The online version contains supplementary material available at 10.1186/s40798-022-00468-1.

## Key Points

- More than half of the Spanish elite track and field female athletes have urinary incontinence.
- Questionnaires, such as 3IQ or ICIQ-UI-SF, could underestimate urinary incontinence prevalence in elite athletes.
- Track and field specialization, years of sports experience, and training volume or resting periods were not related to urinary incontinence.

## Background

Urinary incontinence (UI) is the most prevalent pelvic floor dysfunction (PFD) that occurs as a result of the perineal structure’s inability to cope with an increase in intra-abdominal pressure (IAP) [1, 2]. Despite the multifactorial pathophysiology, physical effort from high-impact sports is important risk factors that could explain a higher prevalence in athletes compared to the sedentary population [3, 4]. Athletes who practice these sports perform continuous ground impacts and/or abdominal contractions that suddenly increase IAP and impact on the pelvic floor (PF) musculature. These physical demands are regularly developed by high-impact athletes specializing in any of the track and field events [5, 6]. Their efforts during training routines may lead to a change in the physiological urethrovesical angle and, thus, urine leakage that may affect their social and personal life [4].

Authors who evaluated UI prevalence in athletes performing in different high-impact sports have confirmed higher UI prevalence for sport modalities that require frequent jumps or vibration due to the high load for the PF [7–9]. When evaluating PF kinematics during sports activities, studies observed increases up to 22.8 kPa in IAP and greater PF deformations during jump-landing [10, 11] or PF movement during running comparable to high-impact activities like coughing [5]. The training routines of athletes specialized in sprint or hurdles events are very different from those of athletes specializing in throwing or jumping events, among others. Although very few studies to date have analyzed the prevalence of UI in track and field athletes [7, 12], authors have shown an especially high prevalence in these events [12]. Sporting activities from track and field could be damaging the perineal structures and facilitating UI, particularly in athletes who periodically perform high-intensity training, such as elite athletes.

Previous studies have suggested that self-reported UI is common among elite and amateur athletes, while objective parameters like urine leakage were higher at the elite level compared to non-elite athlete [13] and much higher than in the nonathletic population [14, 15]. It may be that greater physical exhaustion, physical training volume, and high-intensity training could explain the increased symptomatology for UI in elite athletes [8, 13, 16]. However, no studies to date have taken into account the specific sports tasks that elite track and field athletes perform according to the competitive events for which they are training. For this reason, this study sought to describe the prevalence, type, and severity of self-reported UI among elite male and female track and field athletes, taking into account the events in which they specialize and the training characteristics they have, as well as other risk factors such as demographic variables and medical history.

## Methods

### Design

A cross-sectional study was designed, involving Spanish male and female elite track and field athletes. The STROBE and CHERRIES guidelines were taken into account for this study (Additional files 1 and 2, respectively). The study was conducted according to the Code of Ethics of the World Medical Association (Declaration of Helsinki). The ethical committee of the Universidad Camilo José Cela (Spain) approved this study.

### Participants

During the 2019 outdoor season, all national elite track and field athletes were invited to voluntarily and anonymously participate in this study by the national track and field federation through email. Participants were asked to complete an anonymous online questionnaire through the Survey Monkey^®^ platform (CA, USA). The sample size was calculated as a proportion of finite population, considering a 99% confidence level to estimate an expected prevalence of 50% in the total population, for 5% accuracy in the study. At the time of the study, the National Sports Council certified 711 elite track and field athletes, so the estimated minimum sample size was 345 elite track and field athletes. Following the Spanish Royal Decree 971/2007, elite athletes were defined as those who had a valid certificate of being “high-level athlete” and/or “high-performance athlete.” As inclusion criteria, we considered elite athletes aged 14 years or older, who practice any of the track and field events. We excluded athletes who were pregnant when the study started or in the past year or did not have the ability to adequately understand instructions in Spanish as the national language. Prior to completing the questionnaire, all participants gave their consent to participate after having been informed of the purpose of the study, instructions, and expected time for completing the survey. They were also informed about the research team, location where responses would be stored, time during what data would be stored and data protection (anonymity) issues. Survey data were automatically processed by the platform and stored on an external device with a password-protected server. All information was always in the custody of the research team members, in line with new continental data protection and national norms. Incentives were not offered for participating in the study.

### Patients’ Involvement

Although patients and members of the public were not involved in the design, management, or conduct of the research, once it has been published, participants will be informed of the results through a dedicated website and will be sent details of the results in a study newsletter suitable for a non-specialist audience.

### Questionnaire

The open online questionnaire was designed to be anonymous, and before being spread, usability and proper performance was verified. It was made up of adaptive items, which means that some items were conditionally displayed based on responses to previous items. Thus, total number of pages was variable based on the participants’ responses. To avoid dropouts, the number of items per page ranged from 2 to 6, as maximum. Before the final submission, participants were always able to review and change their responses through a back button. Uncompleted questionnaires were removed from the analysis. It contains four main sections to collect the following information: (a) socio-demographic and anthropometric data: age, sex, weight and height; (b) medical history: common diseases, constipation, urinary infection (urinary tract infection, diagnosed with laboratory tests), gynecological data and problems (i.e., polycystic ovarian syndrome, fibroids, endometriosis, or pelvic inflammatory disease; gynecological disorders related to menstrual disorders, vaginal infections, sexually transmitted diseases, or dyspareunia were analyzed independently), or prostatic problems (i.e., benign prostatic hyperplasia, prostatitis, prostate surgery, or prostate cancer); (c) training characteristics: track and field event specialization (i.e., the main event for which athletes train and compete during the season), years of experience, training volume (hours/day, days/week, weeks/years), and resting (hours between training sessions, weeks/year); (d) UI data: type, severity, and social impact according to International Urogynecological Association and International Continence Society recommendations, as assessed through the following validated questionnaires:

#### International Consultation on Incontinence Questionnaire-UI Short-Form (ICIQ-UI-SF) (Spanish version)

This questionnaire is a self-administered tool to determine if a subject has UI and, if so, its frequency, severity (according to the amount of urine leakage), and whether there are impacts on quality of life (QoL). It consists of 3 items that evaluate these three aspects, respectively. The occurrence of UI is established according to the response to items 3, 4, or 5; when the sum of values from these questions is ≥ 1, UI is considered to be present. The total score is the result of the sum of these 3 items, ranging from 0 to 21 points. Apart from these 3 items, the questionnaire contains 8 additional questions related to the type of UI; these questions are not part of the questionnaire score, but have a descriptive and guiding purpose for assessing the UI type [17].

#### Three Incontinence Questions Questionnaire (3IQ) (Spanish version)

Three questions are used to define whether a subject has had UI in the last 3 months and its type [stress urinary incontinence (SUI), urge urinary incontinence (UUI), or mixed urinary incontinence (MUI)] according to the trigger situation (stress, urge, or no physical activity) [18, 19]. The UI type is defined according to the responses to question 3, with the following possibilities and interpretation: A, “you leak urine most often when you were engaged in physical activity (e.g., coughing, sneezing, lifting, exercising)” (SUI or predominantly SUI); B, “you leak urine most often when you had the urge or feeling that you needed to empty your bladder but you could not get to the toilet fast enough” (UUI or predominantly UUI); C, “you leak urine most often without physical activity and without a sense of urgency” (other causes or predominantly other causes); or D, “you leak urine about equally as often with physical activity as with a sense of urgency” (MUI) [18].

#### Incontinence Severity Index (ISI) (Spanish version)

This consists of two questions about the subject’s experienced frequency of urine leakage (5 levels) and how much urine is lost (4 levels) described as none, drops, small splashes, or more [20]. This incontinence severity is based on data after multiplying the results of the two questions, as follows: slight (score 1–2), moderate (score 3, 4 or 6), severe (score 8–9), or very severe (score 12) [21].

For athletes’ specialization, track and field events were grouped according to the main sport activity required. For the classification, Olympic events were considered as follows: sprint/hurdles (100 m, 200 m, 400 m, 100 m hurdles [mh], 110 mh, 400 mh, and relays); middle-distance running (800 m, 1500 m, 3000 m, and 3000 m steeplechase); long-distance running (> 3000 m); athletic walk (20 and 50 km); horizontal jumps (long jump and triple jump); vertical jumps (high jump and pole vault); throws (discus, javelin, hammer, and shot put); and combined events (decathlon).

### Statistical Analysis

All statistical tests were performed using the package IBM SPSS Statistics v.26.0 (New York, USA). Data are provided as the mean and standard deviation along with 95% confidence intervals (95%CI). When appropriate, data are provided as percentages. The Shapiro–Wilk test was used to check the normality of the data. Between sexes, means were compared using the Student's t test and proportions by the Chi-squared test. The estimated odds ratio (OR) and 95%CI for the OR were analyzed through the risk estimate of crosstabs. One-way ANOVA was used to test the profile of the values, depending on the track and field events in which they are specialized. Bivariate correlations among quantitative variables (demographic, training characteristics, number of pregnancies, and ICIQ-UI-SF) were assessed through Pearson’s coefficient. The level of confidence was set at 95% and significance at *p* < 0.05.

## Results

### Demographic Data, Medical History, and Training Characteristics

Of the invited athletes, 389 gave their consent to participate and accessed the questionnaire (participation rate: 100%); 50 of these 389 athletes did not complete the questionnaire due to personal reasons, so the final sample for the analysis consisted of 339 elite athletes (completion rate: 87.1%; female: 62.2%). Demographic variables and those related to medical history and training characteristics can be found in Table 1. Female athletes showed higher frequencies of urinary infection than males and were more likely to experience gynecological problems than males were to experience prostate problems (*p* < 0.001); none of the male athletes reported prostate surgery or prostate cancer. The rest of the data referring medical history and training characteristics did not show any significant differences (*p* > 0.05). No significant associations were observed between ICIQ-UI-SF and age, BMI, or variables regarding training (*p* > 0.05) (Additional file 3).

**Table 1:** Athletes’ demographic data, medical history, and training characteristics

Variable	Males (*n* = 128)			Females (*n* = 211)			*p* Value
Demographic data	Mean	SD	95%CI	Mean	SD	95%CI	
Age (years)	22.38	5.59	(21.40–23.36)	23.15	5.74	(22.37–23.93)	0.22
Weight (kg)	72.12	14.81	(69.57–74.75)	59.37	11.20	(57.85–60.89)	< 0.001
BMI (kg/m^2^)	22.45	3.86	(21.77–23.12)	20.98	3.27	(20.54–21.43)	< 0.001

Medical history	*n*	%	*n*	%	*p* Value
*Urinary tract infections*					
Yes	15	11.7%	110	52.0	< 0.001
No	113	88.3%	101	48.0	
*Constipation*					
Yes	12	9.4%	34	16.1	0.08
No	116	90.6%	177	83.9	
*Gynecological/prostate problems*					
Yes	2	1.6%	40	19.0	< 0.001
No	126	98.4%	171	81.0	
*Pregnancies*					
Yes	—	—	17	8.1	—
No	—	—	194	91.1	

				Mean	SD	95%CI	*p* Value
Total pregnancies (n)	—	—		1.11	0.48	(0.86–1.36)	—
Vaginal deliveries (n)	—	—		0.75	0.77	(0.33–1.16)	—

Training characteristics	Mean	SD	95%CI	Mean	SD	95%CI	*p* Value
*Training volume*							
Hours/day	2.42	0.82	(2.24–2.59)	2.42	0.74	(2.30–2.55)	0.64
Days/week	4.99	1.26	(4.72–5.25)	5.27	1.08	(5.09–5.46)	0.15
Months/year	10.02	1.15	(9.81–10.23)	10.04	1.63	(9.80–10.27)	0.82
*Resting period*							
Hours between training sessions	20.11	7.00	(18.64–21.59)	18.77	9.25	(17.18–20.36)	0.69
Weeks/year	3.72	2.57	(3.25–4.20)	4.17	2.40	(3.82–4.52)	0.11

*BMI* body mass index, *CI* confidence interval, *SD* standard deviation

For female athletes, when data were analyzed according to track and field events, results showed significant differences in anthropometric variables, with higher weight (*p* < 0.001) for female athletes specialized in throwing (73.02 ± 15.66 kg) compared to those specializing in long-distance running events (52.00 ± 4.38 kg) and athletic walking (52.01 ± 4.11 kg). Also, significant differences were found regarding training volume in terms of days/week (F[7,201] = 2.14, *p* < 0.05) and resting periods in terms of hours/training session (F[7,183] = 2.33, *p* < 0.05) and weeks/year (F[7,178] = 2.84, *p* < 0.05): post hoc analysis showed that female athletes specializing in vertical jump events had significantly lower training volume in terms of days/week compared to those specializing in long-distance running events (*p* = 0.027); and female athletes specializing in horizontal jump events had significant longer resting periods in terms of weeks/year compared to those specializing in sprint/hurdles (*p* = 0.020), middle-distance running (*p* = 0.007), long-distance running (*p* = 0.026), and throwing events (*p* = 0.005). Any other difference was shown for the rest of variables according to the track and field events in which the athletes specialized (*p* > 0.05) (Additional file 4).

For males, we did not find any significant differences according to track and field event specialization (*p* > 0.05).

### Urinary Incontinence Prevalence, Type, and Severity

**Fig. 1:**
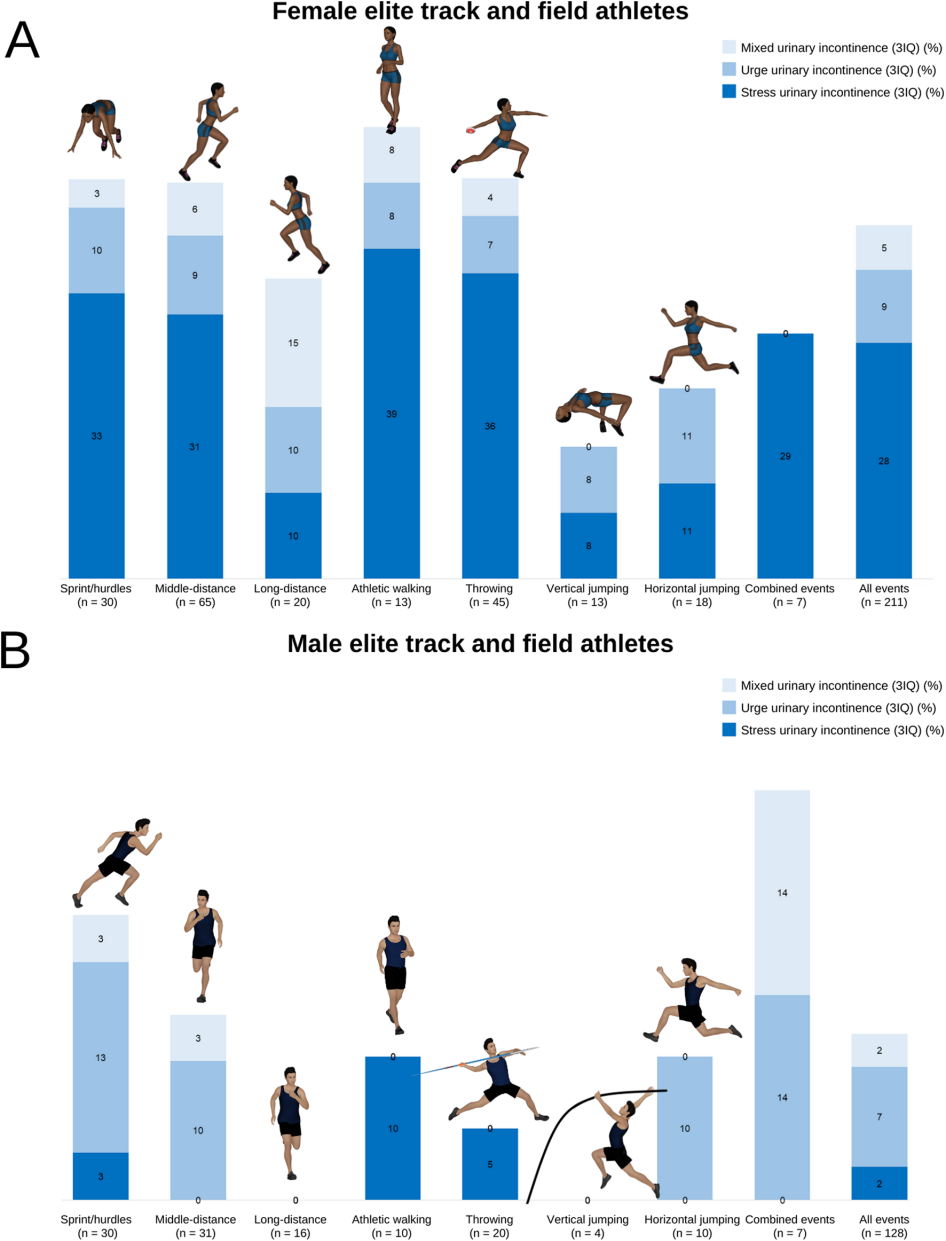
Urinary incontinence prevalence according to ICIQ-UI-SF and 3IQ among elite female and male athletes according to their track and field event specialization

For female athletes, based on ICIQ-UI-SF scores, the overall prevalence of UI was 51.7% (*n* = 109). According to responses to 3IQ, SUI was the most frequent type (64.4%), whereas UUI and MUI accounted for 20.0% and 12.2%, respectively. The ISI scores indicated that, among athletes with UI, 66.7% female athletes described their condition as slight, 28.9% as moderate, and 4.4% as severe. Of female athletes with UI identified by ICIQ-UI-SF, 78.0% (*n* = 85) stated that they had experienced urine leakage during their sport training: 50.6% mainly due to jumping tasks, 16.5% due to running tasks, 8.2% due to powerlifting tasks, and 24.7% due to combined exercises. Of the female athletes who indicated they did not have UI in the ICIQ-UI-SF, 24.6% stated they did urine leakage during training. Female athletes with UI according to ICIQ-UI-SF showed similar training characteristics to the other female athletes (*p* > 0.05), with no significant correlations between the variables for training characteristics and ICIQ-UI-SF score (*p* > 0.05). When these data were analyzed according to track and field event specialization, female athletes specializing in vertical jumps showed a significantly lower UI prevalence compared to those specializing in horizontal jumps (*χ*^2^ [1] = 4.409; *p* = 0.040; OR = 5.238, IC95%: 1.057–25.966), middle-distance running (*χ*^2^ [1] = 4.523; *p* = 0.033; OR = 4.138, IC95%: 1.041–16.444), and sprint/hurdles events (*χ*^2^ [1] = 4.113; *p* = 0.043; OR = 4.359, IC95%: 0.994–19.121) (Fig. 1). No differences were shown for the remaining track and field events (*p* > 0.05).

For male athletes, based on ICIQ-UI-SF scores, the overall prevalence of UI was 18.8% (*n* = 24), which was significantly lower than for female athletes (*χ*^2^ [1] = 36.19; *p* < 0.001). Concerning types of UI based on the responses to 3IQ, UUI predominates (52.9%), while SUI and MUI accounted for 17.6% and 11.8%, respectively. The ISI scores indicated that, among male athletes with UI, 82.4% described their condition as slight, 17.6% as moderate, and none as severe. From male athletes with UI according to ICIQ-UI-SF, 37.5% (*n* = 9) stated experiencing urine leakage during their sport training, mainly due to running tasks (44.4%), due to powerlifting tasks (22.2%), or due to combined exercises (33.4%). Of the male athletes who indicated they did not have UI in the ICIQ-UI-SF, 13.6% stated they did experience urine leakage during training. We did not find any significant differences in the training characteristics of male athletes with and without UI according to ICIQ (*p* > 0.05) or track and field events (*p* > 0.05).

## Discussion

The main findings in the present study showed that more than half of the elite female athletes evaluated had UI according to ICIQ-IU-SF (51.7%), with SUI being the most prevalent type (64.4%) and slight the most frequent level of severity. When we observed data from female athletes who affirmed experiencing urine leakage during their training sessions, this prevalence increased to 78.0%, mainly when performing jumping tasks. In line with this finding, the highest prevalence was observed for female athletes specializing in horizontal jump events followed by those specializing in combined events and sprint/hurdles, but the results were significant only when compared with track and field events that showed lower UI prevalence, such as vertical jump events. As expected, we found significantly lower UI prevalence for male athletes (18.8%), with UUI being the most prevalent UI (52.9%) and without differences according to track and field event specialization.

Our results agree with a previous study that evaluated elite athletes and reported a UI prevalence of more than 45% for female and 14.7% for male athletes. In that study, the authors considered different high-impact sport disciplines, which included track and field [12]. Similarly, a recent meta-analysis estimated that the average prevalence among female athletes was 36.1%, ranging from 19.4 to 76% [22], and the systematic review by Almousa et al. [23] confirmed a 40.6% prevalence among nulliparous female athlete. Although the prevalence reported in these studies was lower than in our results, these studies considered not only elite athletes but also those at different competition levels involving a lower or similar level.

Despite the extended use of the ICIQ-UI-SF, this questionnaire was not specifically developed for elite athletes, and UI prevalence could have been underestimated among our elite athletes. This fact could explain the differences in UI prevalence according to this questionnaire compared with the prevalence according to women who affirmed experiencing urine leakage. Because this population has specific physical demands due to their training sessions, specific tools are needed. This problematic issue has been observed in previous studies, and recently, a subcategory of athletic incontinence has been proposed for SUI related to sports activities or competition [24].

Considering UI types, previous studies also found SUI as the most prevalent UI among female athletes [4] and UUI among male athletes [12]. Rodríguez-López et al. [12] observed a prevalence of 66.0% for SUI in elite female athletes and 38% for UUI in elite male athletes. These data reflect differences for the physiopathology of UI in female and male athletes in elite sport. While SUI is related to physical efforts that increase IAP during high-impact sports and exceed intra-urethral pressure [25], UUI is related to the sudden, compelling need to urinate, involving separate neurogenic, myogenic, or urotheliogenic hypotheses [26], which may be unrelated to sports activities. These aspects should be considered when designing therapeutic management of UI in female or male elite athletes. Although athletes specializing in jumping showed the highest UI prevalence, these data varied when we distinguished UI according SUI, UUI, or MUI types. Athletic walking was the discipline with greatest SUI percentage in both male (10%) and female (39%) athletes. However, the smaller sample size in this discipline and the similar SUI percentages shown in most other disciplines made the differences not statistically significant. Therefore, we are not able to affirm that sports movements or specific physical demands from athletic walking provoked higher SUI prevalence.

In reference to track and field specialization, horizontal jump events, followed by combined events and sprint/hurdles, showed the highest prevalence for UI among female athletes. We should highlight that these results were obtained after taking into account the influence of anthropometric data and medical history as main risk factors traditionally related to UI. However, these results should be considered with caution due to the very wide 95%CI observed. The only study to date, to our knowledge, that analyzed potential differences according to sport activities also observed higher UI prevalence for disciplines that require sprint and acceleration (71.4%) [12]. It is thus important to take into account that female athletes specializing in events that require sprinting often performed plyometric exercises through horizontal jumping as a key training component. In the literature, jumps are most likely to provoke UI [2, 23, 27–31], as we observed in our study when female athletes were asked for the sport activities most related to their urine leakage events. When jumping, the PF has to cope with internal stress due to the sudden shift in the direction of motion, as well as different body mass velocities. To these demands, it is important to add those PF translations occurring after ground contact, apart from pre-activation and post-activation of the PFM needed to deal with landing [6].

Despite this finding, our results showed that female athletes specializing in vertical jump events had the lowest prevalence of UI. To explain this difference, it is worth noting that vertical jump events, which include high jump and pole vault, are track and field jump events that completely differ from those considered to be “high-impact” jumps [6, 32]. Because the landing phase for vertical jumps is not carried out through the support of body weight on the lower limbs, vertical ground reaction forces are not as high as those produced during horizontal jumps. In contrast, horizontal jump events, which include long jump and triple jump, are characterized by landing on the heel, increasing ground reaction forces up to 16 times the body weight [33, 34]. The same mechanism is produced during plyometric exercises performed by female athletes from track and field events like sprint/hurdles and combined events during their training sessions. This could explain why the differences were not statistically significant among all track and field events, although the UI prevalence was higher.

Another important aspect is that female athletes specializing in vertical jump events also showed the lowest training volume in terms of training sessions per week, although training characteristics did not show significant correlations with urine leakage. The lack of differences could be explained by the high intensity and volume that elite athletes performed each day, independently of their track and field specialization. In elite sports, PF displacements, neuromuscular fatigue, and morphological changes of the PF muscles are common risk factors facing elite female athletes [12, 14, 23, 29, 35, 36]. In male athletes, anatomical differences with female athletes could reduce UI prevalence. Regarding UI prevalence among male athletes, none of the male athletes in our study affirmed having had prostate removal, prostate surgery, or prostate cancer. Because prostatectomy is often a major and important factor to consider, their complaints of urine loss could be directly related to their sports practice. In any case, qualitative or other studies specifically designed to explore the UI risk factors in male athletes are needed.

In our study, we did not observe any significant association between ICIQ-UI-SF score and some of the factors traditionally related to such scores, such as age or BMI. Because we considered elite track and field athletes, the ranges in age and BMI were narrow. Contrary to our results, Whitney et al. found that those athletes who suffered UI according to ICIQ-UI-SF tended to be significantly “older” than athletes without UI. Despite this finding, they did not observe any differences between incontinent and continent athletes in BMI and suggested that high-impact sports, mainly those requiring jumping tasks, were the key factor related to UI prevalence and severity [37].

This study has limitations. First, the use of the ICIQ-UI-SF, 3IQ, and ISI questionnaires to consider diagnosis and types of UI prevented us from checking pain, strength, resistance, and coordination of the PF muscles in elite athletes. It is thus important to consider the high correlation of these questionnaires with urodynamic tests or other urogenital diagnostic like a pad test or voiding diaries [2], apart from the extended use to diagnose UI among athletes. Similarly, the cross-sectional character of our design is very limited in evaluating those factors that could provoke UI even before athletes started to suffer it. Prospective studies to evaluate and monitor the training characteristics of athletes are needed to clarify this relationship. Second, because the ICIQ-UI-SF is not a questionnaire specifically for the elite sport population, this could have affected our results. Although new proposals have already been begun to manage this aspect, future studies should consider designing and validating novelty tools specifically for detecting UI among elite athletes. Third, our study did not reach the sample size previously proposed by only 6 participants, and we should highlight that the sample size calculation was estimated taking into account 99%CI instead of 95%CI, which most similar studies often consider. Our sample sizes were also very different among the track and field events. Finally, because our study evaluated elite track and field athletes, the results should not be extrapolated to other sports populations or to different age ranges.

## Conclusions

More than half of the Spanish national elite track and field female athletes have self-reported UI according to ICIQ-UI-SF, with SUI as the most prevalent type. This prevalence is higher if we consider female athletes with urine leakage during training, especially during jumping tasks. Although training characteristics and track and field specialization are not related to ICIQ-UI-SF scores or urine leakage events, female athletes specializing in vertical jump events showed the lowest UI prevalence and training volume in terms of days of training per week. Values were significantly lower for male athletes, with UUI being the most prevalent type and not correlating with their training characteristics or track and field specialization.

Sports professionals and practitioners should take into account our results to increase UI detection among elite athletes and design a specific approach that considers training routines to make visible, prevent, or improve PFD in this population, especially among elite female track and field athletes. These specific approaches should be designed taking into account training characteristics, such as training volume, level of competition, and the physical demands of the sport practice. Factors traditionally related to UI, such as age, should be also considered when designing sanitary approaches.

## Supplementary Information

**Additional file 1.** STROBE Checklist for cross-sectional studies.

**Additional file 2.** CHERRIES Checklist.

**Additional file 3.** Data from Pearson correlations (r score) in female athletes.

**Additional file 4.** Training volume of athletes according to their event specialization and sex.

## Data Availability

The datasets used and/or analyzed during the current study are available from the corresponding author on reasonable request.

## Abbreviations

CI: Confidence interval
IAP: Intra-abdominal pressure
ICIQ-UI-SF: International Consultation on Incontinence Questionnaire-UI Short-Form
ISI: Incontinence severity index
MUI: Mixed urinary incontinence
UI: Urinary incontinence
PF: Pelvic floor
OR: Odds ratio
QoL: Quality of Life
SUI: Stress urinary incontinence
UUI: Urge urinary incontinence
3IQ: Incontinence questionnaire
