# Supplementary material for: Urinary Incontinence Among Elite Track and Field Athletes According to Their Event Specialization: A Cross-Sectional Study
Source: Sports Med Open. 2022 Jun 15;8:78. doi: 10.1186/s40798-022-00468-1 (PMC9200916; doi:10.1186/s40798-022-00468-1)
Supplement: Supplementary file 3 — Additional file 3. Data from Pearson correlations (r score) in female athletes. [file 40798_2022_468_MOESM3_ESM.docx]

**Supplemental file 3**

**URINARY INCONTINENCE AMONG ELITE TRACK AND FIELD ATHLETES ACCORDING TO THEIR EVENT SPECIALIZATION: A CROSS-SECTIONAL STUDY**

**Journal: SPORTS MEDICINE – OPEN**

Rodríguez-López Elena Sonsoles^a^, Acevedo-Gómez María Barbaño^b^, Romero-Franco Natalia^c,*^, Basas-García Ángel^d^, Ramírez-Parenteau Christophe^e^, Calvo-Moreno Sofía Olivia^f^, Fernández-Domínguez Juan Carlos^g^

1. Physiotherapy Department, Universidad Camilo José Cela, E- 28692, Madrid, Spain. E-mail: esrodriguez@ucjc.edu
2. Physiotherapy Department, Spanish Triathlon Federation, Madrid, Spain. E-mail: acevedogomez.maria@gmail.com
3. Nursing and Physiotherapy Department, University of the Balearic Islands, E-07122, Palma de Mallorca, Spain; Health Research Institute of the Balearic Islands (IdISBa). E-mail: narf52@gmail.com
4. Physiotherapy Department, Royal Spanish Athletics Federation, Madrid, Spain. E-mail: abasas@rfea.es
5. Medical Department, Royal Spanish Athletics Federation, Madrid, Spain. E-mail: cramirez@rfea.es
6. Physiotherapy Department, Universidad Camilo José Cela, E- 28692, Madrid, Spain. E-mail: socalvo@ucjc.edu
7. Nursing and Physiotherapy Department, University of the Balearic Islands, E-07122, Palma de Mallorca, Spain; Health Research Institute of the Balearic Islands (IdISBa). E-mail: jcarlos.fernandez@uib.es

*Corresponding author: Natalia Romero-Franco. Nursing and Physiotherapy Department, University of the Balearic Islands. Road to Valldemossa, km 7.5, E-07122, Palma de Mallorca Spain. Twitter handle: @NRomeroFranco; E-mail: [narf52@gmail.com](mailto:narf52@gmail.com)

| **Table 1S. Pearson correlations (r score) in female athletes** | | | | | | | |
| --- | --- | --- | --- | --- | --- | --- | --- |
|  | Age | BMI | Pregnancies (n) | Training (hrs/day) | Training (days/week) | Training (month/year) | Leakage urine during training |
| Age |  |  |  |  |  |  |  |
| BMI | n.s |  |  |  |  |  |  |
| Pregnancies (n) | ,519* | -,508* |  |  |  |  |  |
| Training (hrs/day) | n.s | ,205** | n.s |  |  |  |  |
| Training (days/week) | ,194** | n.s | n.s | ,170* |  |  |  |
| Training (months/year) | n.s | n.s | n.s | n.s | ,349** |  |  |
| Leakage urine during training | n.s | n.s | n.s | n.s | n.s | n.s |  |
| ICIQ-UI-SF (score) | n.s | n.s | n.s | n.s | n.s | n.s | ,331** |
| n.s, non-significant; ICIQ-UI-SF, | | | | | | | |
| * p < 0.05 |  |  |  |  |  |  |  |
| ** p < 0.01 |  |  |  |  |  |  |  |

| **Table 2S. Pearson correlations (r score) in male athletes** | | | | | | |
| --- | --- | --- | --- | --- | --- | --- |
|  | Age | BMI | Training (hrs/day) | Training (days/week) | Training (month/year) | Leakage urine during training |
| Years |  |  |  |  |  |  |
| BMI | ,286** |  |  |  |  |  |
| Training (hrs/day) | n.s | ,236** |  |  |  |  |
| Training (days/week) | n.s | -,178* | ,258** |  |  |  |
| Training (months/year) | ,288** | n.s | ,237** | ,315** |  |  |
| Leakage urine during training | n.s | n.s | n.s | n.s | n.s |  |
| ICIQ-UI-SF (score) | n.s | n.s | n.s | n.s | n.s | n.s |
| n.s, non-significant; ICIQ-UI-SF, | | | | | | |
| ** p < 0.01 |  |  |  |  |  |  |
