# Supplementary material for: Urinary Incontinence Among Elite Track and Field Athletes According to Their Event Specialization: A Cross-Sectional Study
Source: Sports Med Open. 2022 Jun 15;8:78. doi: 10.1186/s40798-022-00468-1 (PMC9200916; doi:10.1186/s40798-022-00468-1)
Supplement: Supplementary file 4 — Additional file 4. Training volume of athletes according to their event specialization and sex. [file 40798_2022_468_MOESM4_ESM.doc]

**Supplemental file 4**

**URINARY INCONTINENCE AMONG ELITE TRACK AND FIELD ATHLETES ACCORDING TO THEIR EVENT SPECIALIZATION: A CROSS-SECTIONAL STUDY**

**Journal: SPORTS MEDICINE – OPEN**

Rodríguez-López Elena Sonsoles^a^, Acevedo-Gómez María Barbaño^b^, Romero-Franco Natalia^c,*^, Basas-García Ángel^d^, Ramírez-Parenteau Christophe^e^, Calvo-Moreno Sofía Olivia^f^, Fernández-Domínguez Juan Carlos^g^

1. Physiotherapy Department, Universidad Camilo José Cela, E- 28692, Madrid, Spain. E-mail: esrodriguez@ucjc.edu
2. Physiotherapy Department, Spanish Triathlon Federation, Madrid, Spain. E-mail: acevedogomez.maria@gmail.com
3. Nursing and Physiotherapy Department, University of the Balearic Islands, E-07122, Palma de Mallorca, Spain; Health Research Institute of the Balearic Islands (IdISBa). E-mail: narf52@gmail.com
4. Physiotherapy Department, Royal Spanish Athletics Federation, Madrid, Spain. E-mail: abasas@rfea.es
5. Medical Department, Royal Spanish Athletics Federation, Madrid, Spain. E-mail: cramirez@rfea.es
6. Physiotherapy Department, Universidad Camilo José Cela, E- 28692, Madrid, Spain. E-mail: socalvo@ucjc.edu
7. Nursing and Physiotherapy Department, University of the Balearic Islands, E-07122, Palma de Mallorca, Spain; Health Research Institute of the Balearic Islands (IdISBa). E-mail: jcarlos.fernandez@uib.es

*Corresponding author: Natalia Romero-Franco. Nursing and Physiotherapy Department, University of the Balearic Islands. Road to Valldemossa, km 7.5, E-07122, Palma de Mallorca Spain. Twitter handle: @NRomeroFranco; E-mail: [narf52@gmail.com](mailto:narf52@gmail.com)

**Table S1. Training volume of athletes according to their event specialization and sex.**

|  | **FEMALE** | | | | | **MALE** | | | | |
| --- | --- | --- | --- | --- | --- | --- | --- | --- | --- | --- |
| **Training volume (hrs/day)** | **MEAN** | **SD** | **95% CI** | | **p-value** | **MEAN** | **SD** | **95% CI** | | **p-value** |
| Sprint/hurdles | 2.40 | 0.89 | 2.07 | 2.73 | 0.084 | 2.40 | 0.56 | 2.19 | 2.61 | 0.151 |
| Middle-distance run | 2.37 | 0.81 | 2.16 | 2.57 |  | 2.26 | 0.82 | 1.96 | 2.56 |  |
| Long-distance run | 2.40 | 0.94 | 1.96 | 2.84 |  | 2.56 | 0.96 | 2.05 | 3.08 |  |
| Athletic walking | 2.31 | 0.63 | 1.93 | 2.69 |  | 2.60 | 0.52 | 2.23 | 2.97 |  |
| Throwing | 2.73 | 0.72 | 2.52 | 2.95 |  | 2.75 | 1.02 | 2.27 | 3.23 |  |
| Vertical jumps | 2.46 | 0.66 | 2.06 | 2.86 |  | 1.75 | 0.50 | 0.95 | 2.55 |  |
| Horizontal jumps | 2.33 | 0.49 | 2.09 | 2.57 |  | 2.20 | 0.63 | 1.75 | 2.65 |  |
| Combined events | 3.14 | 1.07 | 2.15 | 4.13 |  | 2.71 | 0.49 | 2.26 | 3.17 |  |
| Total | 2.48 | 0.80 | 2.37 | 2.59 |  | 2.44 | 0.78 | 2.30 | 2.57 |  |
| **Training volume (days/week)** | **MEAN** | **SD** | **95% CI** | | **p-value** | **MEAN** | **SD** | **95% CI** | | **p-value** |
| Sprint/hurdles | 5.17 | 0.91 | 4.83 | 5.51 | 0.041 | 5.17 | 0.75 | 4.89 | 5.45 | 0.010 |
| Middle-distance run | 5.33 | 1.23 | 5.02 | 5.64 |  | 5.19 | 1.28 | 4.73 | 5.66 |  |
| Long-distance run | 5.95 | 1.23 | 5.37 | 6.53 |  | 6.00 | 1.21 | 5.35 | 6.65 |  |
| Athletic walking | 5.46 | 0.78 | 4.99 | 5.93 |  | 5.50 | 0.97 | 4.80 | 6.20 |  |
| Throwing | 5.38 | 0.72 | 5.16 | 5.59 |  | 4.75 | 1.16 | 4.21 | 5.29 |  |
| Vertical jumps | 4.69* | 1.18 | 3.98 | 5.41 |  | 4.25* | 1.50 | 1.86 | 6.64 |  |
| Horizontal jumps | 5.00 | 1.03 | 4.49 | 5.51 |  | 4.40 | 1.51 | 3.32 | 5.48 |  |
| Combined events | 5.00 | 1.41 | 3.69 | 6.31 |  | 4.71 | 1.38 | 3.44 | 5.99 |  |
| Total | 5.31 | 1.08 | 5.16 | 5.45 |  | 5.13 | 1.21 | 4.91 | 5.34 |  |
| **Training volume (months/year)** | **MEAN** | **SD** | **95% CI** | | **p-value** | **MEAN** | **SD** | **95% CI** | | **p-value** |
| Sprint/hurdles | 9.50 | 2.21 | 8.68 | 10.32 | 0.155 | 9.73 | 1.80 | 9.06 | 10.41 | 0.490 |
| Middle-distance run | 10.00 | 1.77 | 9.55 | 10.45 |  | 10.17 | 0.76 | 9.88 | 10.46 |  |
| Long-distance run | 10.85 | 0.81 | 10.47 | 11.23 |  | 10.43 | 1.02 | 9.84 | 11.02 |  |
| Athletic walking | 10.38 | 0.87 | 9.86 | 10.91 |  | 10.20 | 0.79 | 9.64 | 10.76 |  |
| Throwing | 9.93 | 1.45 | 9.48 | 10.38 |  | 9.65 | 1.27 | 9.06 | 10.24 |  |
| Vertical jumps | 9.92 | 1.26 | 9.16 | 10.68 |  | 10.00 | 1.41 | 7.75 | 11.25 |  |
| Horizontal jumps | 9.59 | 1.50 | 8.82 | 10.36 |  | 9.50 | 1.27 | 8.59 | 10.41 |  |
| Combined events | 10.29 | 0.95 | 9.41 | 11.17 |  | 10.14 | 1.46 | 8.79 | 11.50 |  |
| Total | 9.99 | 1.61 | 9.77 | 10.21 |  | 9.95 | 1.29 | 9.72 | 10.18 |  |
| CI, confidence interval; SD, standard deviation. | | | | | | | | | | |
